# Performance Evaluation of Cooperative Driving Automation Services Enabled by Edge Roadside Units

**DOI:** 10.3390/s26020504

**Published:** 2026-01-12

**Authors:** Un-Seon Jung, Cheol Mun

**Affiliations:** Department of Electronic Engineering, Korea National University of Transportation, Chungju 27469, Republic of Korea; usjung@ivh.co.kr

**Keywords:** cooperative driving automation (CDA), vehicle-to-everything (V2X), edge roadside unit (edge RSU), multi-access edge computing (MEC), cooperative sensing, cooperative maneuvering, sensor sharing

## Abstract

Research on Cooperative Driving Automation (CDA) has advanced to overcome the limited perception range of onboard sensors and the difficulty of inferring surrounding vehicles’ intentions by leveraging vehicle-to-everything (V2X) communications. This paper models how an autonomous vehicle receives cooperative sensing and cooperative maneuvering information generated at an edge roadside unit (edge RSU) that integrates roadside units (RSUs) with multi-access edge computing (MEC), and how the vehicle fuses this information with its onboard situational awareness and path-planning modules. We then analyze the performance gains of edge RSU-enabled services across diverse traffic environments. In a highway-merging scenario, simulations show that employing the edge RSU’s sensor sharing service (SSS) reduces collision risk relative to onboard-only baselines. For unsignalized intersections and roundabouts, we further propose a guidance-driven Hybrid Pairing Optimization (HPO) scheme in which the edge RSU aggregates CAV intents/trajectories, resolves spatiotemporal conflicts via lightweight pairing and time window allocation, and broadcasts maneuver guidance through MSCM. Unlike a first-come, first-served (FCFS) policy that serializes passage, HPO injects edge guidance as soft constraints while preserving arrival order fairness, enabling safe concurrent passage opportunities when feasible. Across intersections and roundabouts, HPO improves average speed by up to 192% and traffic throughput by up to 209% compared with FCFS under identical demand in our simulations.

## 1. Introduction

To achieve Level-4 or higher automated driving, recent research has intensified on Automated Driving Systems (ADSs) that integrate advanced perception and AI-based decision-making modules to ensure traffic efficiency and safety. However, relying exclusively on onboard sensors (OBSs) imposes fundamental limitations due to occlusions, restricted sensing ranges, and difficulties in anticipating the intentions of surrounding vehicles. These limitations become critical in urban environments, where non-line-of-sight (NLOS) conditions and high traffic density are pervasive [1].

To overcome these constraints, Cooperative Driving Automation (CDA) has emerged as a promising paradigm that enables information sharing among Connected Automated Vehicles (CAVs), and between CAVs and infrastructure through vehicle-to-everything (V2X) communication [2,3]. In particular, edge roadside units (edge RSUs)—RSUs integrated with multi-access edge computing (MEC)—enable scalable, low-latency data processing and are well-suited to support cooperative perception and maneuvering in complex traffic scenarios [4].

The Society of Automotive Engineers (SAE) codifies the scope and architecture of CDA systems through a family of standards. SAE J3216 defines cooperative functions, roles, and message freshness/QoS requirements [5]. SAE J3224 specifies the sensor sharing service (SSS), in which a CAV or an RSU shares environmental perception via Sensor Data Sharing Messages (SDSMs) [6]. SAE J3186 outlines the Maneuver Sharing and Coordinating Service (MSCS), which distributes vehicle intentions and coordinated guidance using the Maneuver Sharing and Coordinating Message (MSCM) format [7].

Recent research has explored the performance benefits of cooperation via V2X. Some studies report improvements in traffic safety and flow when vehicles exchange sensor or intent information [8,9]. Others emphasize system-level factors such as communication delay and computational latency [10,11]. Field demonstrations and prototypes have validated the feasibility of cooperative perception and maneuver planning, but often without a full-stack evaluation that includes infrastructure-side generation and in-vehicle fusion of cooperative artifacts [12].

For instance, Zainudin et al. [13] simulate sensor sharing in roundabouts using SUMO–CARLA but do not explicitly model how received messages are fused within the CAV’s planning stack. Similarly, Buchholz et al. [14] use edge servers to build an environment model based on infrastructure sensors, but their evaluation focuses on occlusion handling rather than maneuver coordination. Yu et al. [15] present an adaptive fusion network for V2I perception sharing, achieving enhanced 3D object detection, yet their method does not integrate maneuver guidance nor plan-level decision fusion.

Recent studies have also examined CDA under vehicular edge computing (VEC), where cooperative perception and computation are jointly optimized subject to latency and resource constraints. For example, Zhao et al. proposed a multi-agent Deep Reinforcement Learning (DRL) formulation that co-optimizes cooperative perception and computation in VEC to enhance region-of-interest perception while mitigating computational burden [16]. Beyond cooperative perception and computation offloading, recent work has also focused on scalable control architectures for coordinated CAV operation. For example, Liang et al. proposed a multi-agent system (MAS)-based hierarchical cooperation architecture that decomposes cooperative control into layered decision modules, enabling coordination among connected vehicles under heterogeneous objectives and constraints [17]. These architectural trends further motivate infrastructure-assisted CDA, where edge RSUs can provide locally consistent perception/guidance while preserving vehicle autonomy and interpretability.

From the system design perspective, Xu et al. [18] propose OpenCDA, a modular co-simulation framework that supports cooperative lane change and merging. However, their implementation lacks RSU-to-vehicle maneuver guidance and realistic intersection policy evaluation. Coopernaut [19] introduces end-to-end learning for cooperative perception across networked vehicles, but does not model infrastructure-led guidance or formal message standards (e.g., SAE J3224/J3186). Mertens et al. [20] and Guo et al. [21] investigate decentralized and probabilistic coordination methods at intersections, yet these rely on inter-vehicle planning and omit edge coordination logic.

Recent efforts to systematize V2X maneuver coordination—such as Molina-Masegosa et al. [22]—emphasize the challenges in timing, message structure, and role negotiation, but their work is conceptual and lacks integration with a vehicle-level fusion pipeline or trajectory planner. Collectively, these studies demonstrate the growing interest in cooperative automation, yet a consistent gap remains: few works integrate both sensor-level sharing (e.g., SDSM) and maneuver coordination (e.g., MSCM) into a unified CDA system that models message semantics, edge computation, and in-vehicle fusion in diverse traffic environments.

Despite this progress, most existing works either focus on (i) computation/offloading and communication-aware optimization at the edge, or (ii) cooperative perception benefits without explicitly modeling how standardized infrastructure-generated artifacts are fused inside the vehicle stack and injected into onboard planning. This paper fills the gap by modeling a vehicle-side integrated decision process that fuses SAE-compliant SDSM (SSS) and RSU-generated MSCM guidance (MSCS) with onboard sensing and trajectory planning, enabling a full-stack performance evaluation across merges, unsignalized intersections, and roundabouts.

### Contributions

This paper addresses the integration of edge infrastructure–vehicle cooperation into a cohesive CDA framework. In contrast to existing studies that either focus solely on cooperative perception [13,14,15] or decentralized maneuver coordination among CAVs [20,21], our work unifies both aspects—edge-generated sensing and guidance—into a full-stack simulation and vehicle-side fusion pipeline. Our key contributions are summarized as follows:

- Edge RSU system and interfaces: We formalize a roadside infrastructure system composed of RSUs integrated with MEC. This system generates cooperative sensing and maneuver coordination artifacts compliant with SAE J3216 CDA service definitions, specifically the SSS [6] and the MSCS [7]. We detail the message formats (SDSM, MSCM) and V2X PC5-based dissemination logic for interaction with CAVs.
- Vehicle-side integrated decision model: We propose a perception-and-planning fusion pipeline that jointly integrates (i) edge-sourced sensor information (SDSM), (ii) maneuver guidance (MSCM), and (iii) onboard sensing (OBS). Unlike prior work such as OpenCDA [18] and Coopernaut [19], which lack infrastructure-sourced maneuver integration, our system explicitly models decision fusion using Dynamic Occupancy Grid Maps (DOGMs) and trajectory planning in the Frenet frame. This extends studies such as Zainudin et al. [13] and Buchholz et al. [14], where infrastructure sensing is used but onboard fusion is limited or omitted.
- Multi-scenario simulation and performance evaluation: We implement our CDA framework in a co-simulation environment composed of MATLAB R2024b Automated Driving Toolbox, MATLAB Driving Scenario Designer, and V2X message emulation. We evaluate performance across three representative urban traffic scenarios—highway merging, unsignalized intersections, and roundabouts—using metrics such as average vehicle delay and throughput. Our findings show the following:
  – Edge-enabled SSS improves safety in occlusion-heavy configurations compared to onboard-only sensing.
  – MSCS-based cooperative maneuvering via our proposed Hybrid Pairing Optimization (HPO) scheme outperforms conventional first-come, first-served (FCFS) control in both average delay and traffic throughput.
  These results validate the synergistic benefits of cooperative perception and maneuver sharing in realistic, safety-critical scenarios.
- Theoretical generalization and deployment implications: While most prior studies assess either vehicle-only coordination [21,22] or abstract message-passing algorithms, we model full-stack RSU-to-vehicle integration. Our framework supports infrastructure role modeling [22] and internal–external fusion reliability. It also provides deployment insights applicable to smart intersection pilot deployments, hybrid traffic environments, and SAE-compliant message standardization efforts.

To the best of our knowledge, this is the first study to implement and evaluate a full-stack CDA system that integrates both SSS and MSCS from edge RSUs into onboard planning logic, validated across diverse conflict-rich traffic scenarios with explicit message and decision fusion modeling.

The remainder of this paper is organized as follows. Section 2 presents the edge RSU architecture and message semantics. Section 3 describes the vehicle-side fusion pipeline. Section 4 reports scenario-based simulation results and performance analysis. Section 5 discusses deployment considerations. Section 6 concludes with deployment implications and future work.

## 2. Edge RSU Model and Message Interfaces

This section specifies the functional architecture of the edge RSU and its message interfaces to CAVs, in alignment with SAE J3216 (cooperative automation roles and QoS), SAE J3224 (SSS and SDSM), and SAE J3186 (MSCS and MSCM). Edge RSU combines roadside perception sensors with MEC to generate cooperative situational awareness and maneuvers, which are deployed to CAVs via the V2X PC5 interface.

### 2.1. Functional Architecture

**Figure 1:**
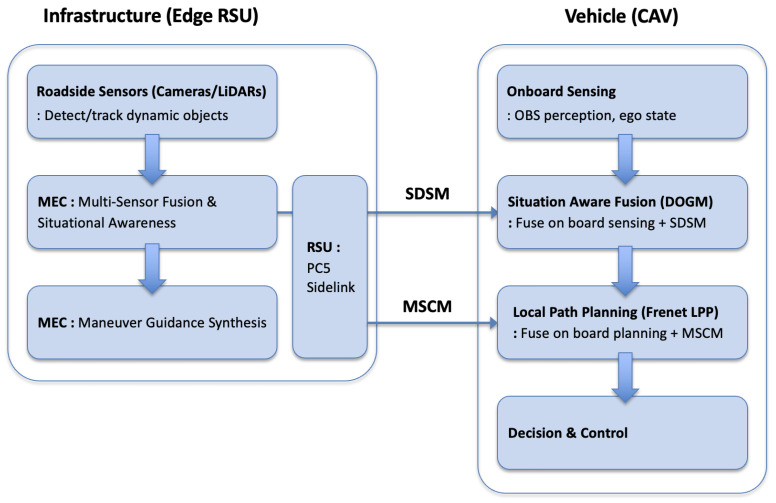
Edge RSU model and interfaces.

Figure 1 illustrates the proposed stack. Roadside sensors (e.g., cameras or LiDARs) are mounted near conflict zones such as merges, unsignalized intersections, and roundabouts. These sensors detect and track dynamic objects—vehicles, two-wheelers, and pedestrians—and produce object state estimates. Sensor outputs are streamed to the MEC node, which executes (i) multi-sensor fusion and situation awareness and (ii) maneuver guidance synthesis based on roadway context and CAV intents. The MEC serializes its outputs into (a) SDSM s (perception sharing; SAE J3224) and (b) MSCMs (maneuver coordination; SAE J3186).

The RSU radio unit provides PC5 PHY/MAC support, GNSS-disciplined timing, and security primitives. These SDSM and MSCMs are broadcast over PC5 sidelink to nearby CAVs. CAVs concurrently transmit their own observations and planned intents to close the cooperative loop. Each vehicle executes onboard perception, planning, and decision logic, supplemented by a timing-aware fusion module that integrates SDSM and MSCM inputs with its own OBS outputs.

### 2.2. Cooperative Perception Pipeline—SSS/SDSM (SAE J3224)

Cooperative perception extends a CAV’s effective field of view beyond onboard sensing (OBS) limitations due to line-of-sight (LoS) constraints and mitigates occlusions by disseminating infrastructure-fused object states. The MEC first performs multi-view association and track-to-track fusion; contextual inference covers lane topology, right-of-way assignment, blind-spot occupancy, and residual risk scoring.

Subsequently, in accordance with SAE J3224, the MEC serializes the fused perception data into the following SDSM structure:

- Header: Station ID (edge RSU), GNSS timestamp, RSU pose.
- Frames: East-North-Up (ENU) map frame, including alignment hints for transformation into the vehicle coordinate frame.
- Object list: Object ID i∈{1,…,N}, object state ${\hat{o}}_{i}\left(t\right)$.

The object state of object *i* is defined as

(1) $${\hat{o}}_{i}\left(t\right)=\left\{x_{i}\left(t\right),y_{i}\left(t\right),v_{i}\left(t\right),\theta_{i}\left(t\right),\Sigma_{i}\left(t\right),c_{i},a_{i}\right\},$$

where $\left(x_{i}\left(t\right),y_{i}\left(t\right)\right)$ denote map-fixed spatial coordinates, $v_{i}\left(t\right)$ is the velocity vector, $\theta_{i}\left(t\right)$ the heading angle, $\Sigma_{i}\left(t\right)$ the covariance matrix, $c_{i}$ the object class label, and $a_{i}$ the Age of Information (AoI) indicating the staleness of the estimate.

Each SDSM at time *t* aggregates the set of all *N* tracked objects as

(2) $$\mathrm{SDSM}\left(t\right)=\{{\hat{o}}_{i}\left(t\right)|i=1,\ldots ,N\}.$$

Finally, the RSU periodically broadcasts SDSMs over PC5 sidelink. CAVs may also report their OBS-derived object detections over PC5 to close the cooperative sensing loop.

### 2.3. Cooperative Maneuvering Pipeline—MSCS/MSCM (SAE J3186)

Cooperative maneuvering resolves short-horizon conflicts and coordinate flows at merges and intersection-like geometries via edge-generated advisories. The MEC aggregates PC5 sidelink intents (trajectory snippets, desired gap/speed, priority flags) and synthesizes guidance using a policy module (e.g., HPO). Safety filters and comfort constraints (acceleration/jerk) ensure feasibility. Compliant with SAE J3186, the MEC serializes coordinated guidance into the following:

- Header: Station ID, timestamp, scene/zone ID.
- Per-CAV guidance: Lane/slot and sequence, reference path primitive or short trajectory, speed window $\left[v_{\min},v_{\mathrm{ref}},v_{\max}\right]$, time-to-enter/leave windows.

MSCM is broadcast on PC5 sidelink event-triggered with periodic refresh; CAVs acknowledge or update intents in Time Division Duplexing (TDD) mode on PC5 sidelink, closing the loop.

## 3. Vehicle-Side Fusion Pipeline

**Figure 2:**
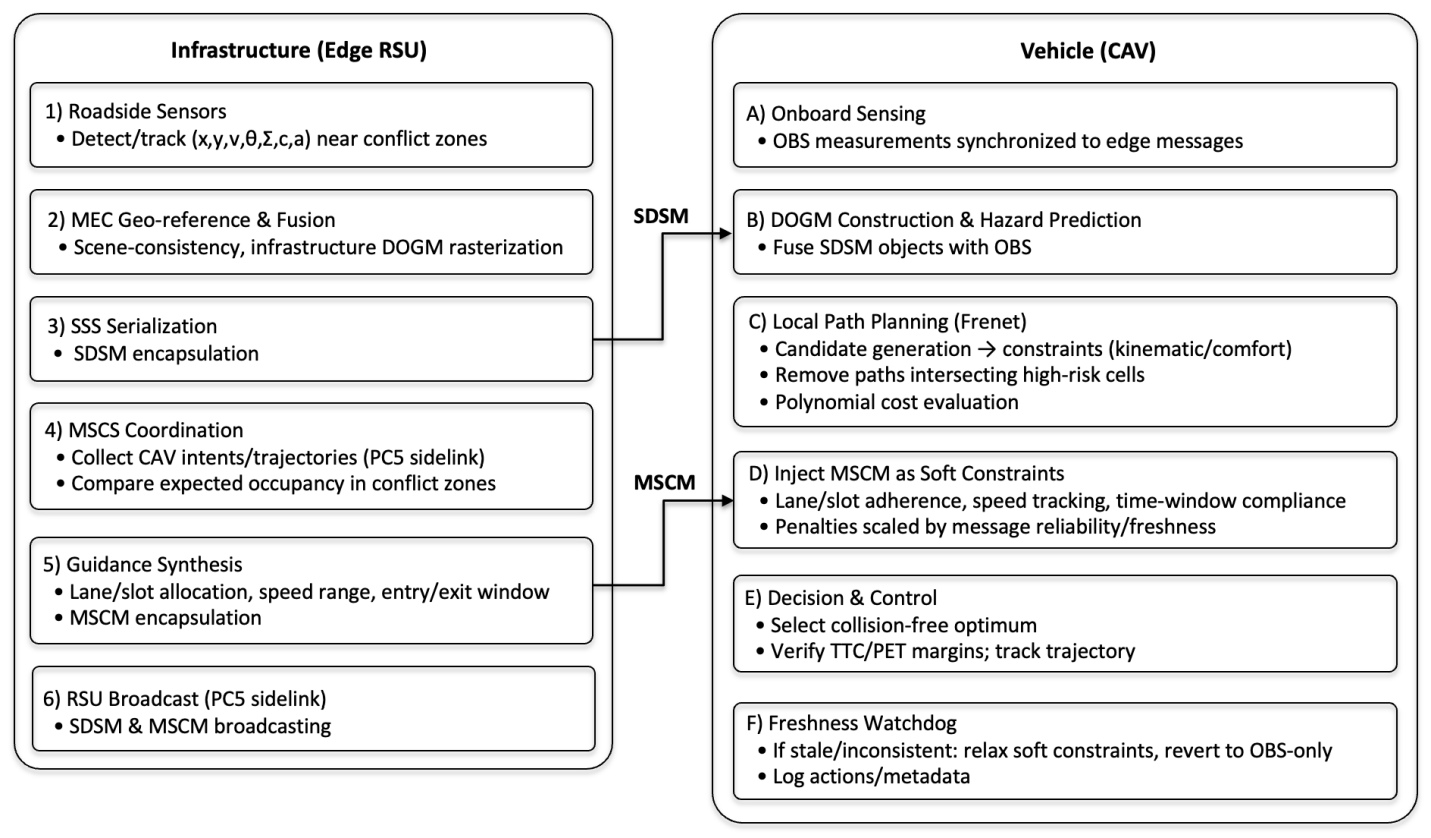
Cooperative operation flow (edge RSU ↔ CAV).

This section details the vehicle-side pipeline that integrates edge-generated cooperative messages—perception (SDSM) and maneuver guidance (MSCM)—with onboard sensing and planning. Perception is represented by a DOGM [23,24,25]; cooperative guidance is embedded into a Frenet-based local path planning (LPP) module [26]. The proposed architecture in Figure 2 visualizes the novel coordination of perception and guidance modules through SDSM and MSCM fusion, offering a comprehensive decision-making flow inside the CAV.

### 3.1. Pipeline Overview via Operation Flow

Figure 2 visualizes the end-to-end cooperative loop between edge infrastructure and vehicles. On the infrastructure side, roadside sensors (e.g., cameras/LiDARs) are first geo-referenced and fused at the MEC to generate scene consistency estimates for dynamic objects and free space. This fused situational awareness data is rasterized into an infrastructure DOGM and serialized into SDSMs for the SSS. Simultaneously, the MEC acts as the MSCS coordinator: it collects maneuver intent/trajectory snippets sent from nearby CAVs via the PC5 sidelink, compares expected occupancy in conflict zones (merges, signal-free intersections, rotaries), and synthesizes vehicle-specific guidance (lane/slot allocation, recommended speed range, entry/exit time window) encapsulated in MSCMs. Both SDSM and MSCM are broadcast to all CAVs within the RSU service area via the PC5 sidelink.

The vehicle provides raw observations synchronized with the received edge message via onboard sensors. The vehicle constructs its own DOGM and generates predicted hazard regions on the local map by fusing SDSM objects with OBS evidence using weights that account for the recency and quality of the information. Subsequently, the local path planner operates in the Frenet coordinate system: it generates candidate trajectories, applies kinematic/comfort constraints, removes paths intersecting high-risk cells, and evaluates the remaining set using a polynomial cost function. MSCM directives are injected as soft constraints such as lane/slot adherence, speed tracking, and time window compliance. Penalties are adjusted based on message reliability and freshness, ensuring cooperation remains recommended even under uncertainty. The planner selects the optimal collision-free trajectory, the supervisory decision layer verifies safety margins (e.g., Time to Collision (TTC)/Post-Encroachment Time (PET) buffers), and the low-level controller executes commands. If the message freshness threshold is violated or inconsistencies occur, the vehicle flexibly mitigates the guidance cost and reverts to an OBS-only policy until new SDSM/MSCM arrives. Resulting actions and message metadata are logged, and this loop repeats each control cycle, completing the cooperative perception–planning–control loop shown in the figure.

### 3.2. Perception Fusion with a Dynamic Occupancy Grid Map

To robustly fuse edge-generated SDSM with OBS in cluttered, partially occluded scenes, we adopt a DOGM as the unified world representation. A DOGM discretizes the 2D space into small cells and jointly estimates, per cell, (i) the occupancy probability and (ii) the dynamic state (e.g., velocity), enabling consistent filtering of heterogeneous measurements and short-horizon motion prediction.

Let cell c∈C cover area $A_{c}$. The cell state at time *t* is

(3) $$s_{c}\left(t\right)=\left[p_{c}^{\mathrm{occ}}\left(t\right),\;v_{c}\left(t\right),\;\Sigma_{c}^{v}\left(t\right)\right],$$

where $p_{c}^{\mathrm{occ}}\left(t\right)\in \left[0,1\right]$ is the occupancy probability, $v_{c}\left(t\right)={\left[v_{c}^{x}\left(t\right),v_{c}^{y}\left(t\right)\right]}^{⊤}$ is the cell-wise velocity, and $\Sigma_{c}^{v}\left(t\right)$ is its velocity covariance.

To enable stable Bayesian fusion, we maintain occupancy in log-odds form. To prevent divergence when $p_{c}^{\mathrm{occ}}\left(t\right)$ approaches 0 or 1, we first clip the probability using

(4) $${\tilde{p}}_{c}^{\mathrm{occ}}\left(t\right)=\min\left(\max\left(p_{c}^{\mathrm{occ}}\left(t\right),\varepsilon \right),1-\varepsilon \right),$$

where ε is a small positive constant (e.g., $\varepsilon =10^{-6}$). The log-odds value is then computed using the natural logarithm

(5) $$L_{c}\left(t\right)=\ln\left(\frac{{\tilde{p}}_{c}^{\mathrm{occ}}\left(t\right)}{1-{\tilde{p}}_{c}^{\mathrm{occ}}\left(t\right)}\right),$$

and its inverse transformation yields the occupancy probability

(6) $${\tilde{p}}_{c}^{\mathrm{occ}}\left(t\right)=\frac{1}{1+\exp\left\{-L_{c}\left(t\right)\right\}}.$$

This log-odds representation ensures numerically stable fusion of heterogeneous sensor observations while maintaining boundedness and consistency during updates.

At each planning tick $t_{k}$, measurements arrive from (i) OBS (e.g., camera/LiDAR/Radar) and (ii) edge RSU SDSM objects ${\left\{{\hat{o}}_{i}\right\}}_{i=1,\ldots ,N}$. Both are mapped to cell-level inverse sensor models $m_{c}^{\mathrm{OBS}}\left(t_{k}\right)$ and $m_{c}^{\mathrm{SDSM}}\left(t_{k}\right)$ that provide occupied/free evidence and, when available, velocity votes for the traversed cells. With an independent-evidence assumption at the cell level, the log-odds update is

(7) $$L_{c}\left(t_{k}\right)=L_{c}\left(t_{k}^{-}\right)+\lambda_{\mathrm{obs}}\;\ell \left(m_{c}^{\mathrm{OBS}}\left(t_{k}\right)\right)+\lambda_{\mathrm{sdsm}}\;{\bar{w}}_{c}^{\mathrm{SDSM}}\;\ell \left(m_{c}^{\mathrm{SDSM}}\left(t_{k}\right)\right),$$

where the cell-level occupancy belief $L_{c}\left(t_{k}\right)$ is updated by incorporating log-likelihood contributions from both onboard sensing (OBS) and edge-generated cooperative perception (SDSM). The term $\ell (m_{c}\left(t_{k}\right))$ denotes the log-likelihood contribution of a new observation $m_{c}\left(t_{k}\right)$, computed using the inverse sensor model for cell *c*. Let o∈{0,1} be a binary variable indicating the cell’s occupancy state, where o=1 denotes occupied and o=0 denotes free. Then the log-likelihood contribution is defined as

(8) $$\ell \left(m_{c}\left(t_{k}\right)\right)=\ln\left(\frac{p\left(m_{c}\left(t_{k}\right)|o=1\right)}{p\left(m_{c}\left(t_{k}\right)|o=0\right)}\right),$$

quantifying the strength of evidence favoring occupancy over free space. While $L_{c}\left(t_{k}\right)$ maintains a persistent memory state over time, ℓ(·) provides a transient update derived from instantaneous sensor measurements.

In Equation (7), we explicitly separate modality-level trust from object-/cell-level trust. The scalar gains $\lambda_{\mathrm{obs}}$ and $\lambda_{\mathrm{sdsm}}$ weight the overall influence of (i) OBS and (ii) infrastructure-generated V2X perception delivered via SDSM, respectively. These gains represent policy parameters configured at the vehicle stack (or by the system designer) to reflect deployment-dependent factors such as sensing geometry, expected occlusion patterns, V2X link reliability/latency budgets, and the intended cooperation mode. In contrast, $w_{ci}^{\mathrm{SDSM}}$ (defined below) is an object-/cell-level confidence weight that modulates each SDSM object *i* contributing to grid cell *c* using object-specific freshness (AoI) and estimation uncertainty (covariance). Therefore, $\lambda_{\left\{\cdot \right\}}$ and $w_{ci}^{\mathrm{SDSM}}$ capture different sources of uncertainty and are not redundant: $\lambda_{\left\{\cdot \right\}}$ encodes source-level (OBS vs. SDSM) trust, while $w_{ci}^{\mathrm{SDSM}}$ encodes instance-level (object-/cell-level) trust within the SDSM payload. In our implementation, $\lambda_{\mathrm{obs}}$ and $\lambda_{\mathrm{sdsm}}$ are selected by a policy that reflects the intended operating regime. A typical and interpretable policy is to increase reliance on SDSM at long range and/or under occlusions, while prioritizing OBS at short range. This policy operationalizes the design rationale that SDSM is most beneficial for extending perception beyond OBS visibility (e.g., NLOS regions and long-range cells), whereas OBS typically provides higher fidelity in the near field.

To incorporate quality-aware weighting of SDSM evidence, each object $i\in O_{c}$ contributing to cell *c* is assigned a weight $w_{ci}^{\mathrm{SDSM}}\in \left[0,1\right]$ based on its AoI $a_{i}$ and covariance matrix $\Sigma_{i}$. The weight is given by

(9) $$w_{ci}^{\mathrm{SDSM}}=\frac{1}{1+\alpha a_{i}}\cdot \left(\frac{1/\mathrm{tr}(\Sigma_{i})}{1/\mathrm{tr}(\Sigma_{i})+\kappa }\right),$$

where α,κ>0 are tunable parameters that map information staleness and uncertainty into bounded weights. This formulation ensures freshness- and uncertainty-aware fusion consistent with SAE J3216 timing and QoS semantics. We define the average SDSM evidence weight for the log-odds update as

$${\bar{w}}_{c}^{\mathrm{SDSM}}=\frac{1}{|O_{c}|}\sum_{i\in O_{c}}w_{ci}^{\mathrm{SDSM}}.$$

For velocity estimation, cell-level velocity $v_{c}\left(t_{k}\right)$ is updated from both OBS flow cues and SDSM-projected kinematics as

(10) $$v_{c}\left(t_{k}\right)={\left(\Sigma_{c}^{v^{-1}}\left(t_{k}^{-}\right)+\sum_{r\in R_{c}}\Sigma_{r}^{-1}+\sum_{i\in O_{c}}w_{ci}^{\mathrm{SDSM}}\Sigma_{i}^{-1}\right)}^{-1}\left(\Sigma_{c}^{v^{-1}}\left(t_{k}^{-}\right)v_{c}\left(t_{k}^{-}\right)+\sum_{r\in R_{c}}\Sigma_{r}^{-1}v_{r}+\sum_{i\in O_{c}}w_{ci}^{\mathrm{SDSM}}\Sigma_{i}^{-1}v_{i}\right),$$

where $R_{c}$ and $O_{c}$ index OBS- and SDSM-derived velocity contributions to cell *c*. We apply this update only when $p_{c}^{\mathrm{occ}}\left(t_{k}\right)\geq \tau_{\mathrm{occ}}$ to suppress noise from unoccupied cells.

Each SDSM ${\left\{{\hat{o}}_{i}\right\}}_{i=1,\ldots ,N}$ is rasterized into the DOGM along its footprint and projected motion, contributing both occupancy and velocity votes with the weight $w_{c}^{\mathrm{SDSM}}$ and $w_{ci}^{\mathrm{SDSM}}$. In merges/intersections/roundabouts, SDSM contributions are expanded along admissible lanes from the HD map to reflect likely paths. The planner consumes (i) clustered dynamic obstacles with covariances and (ii) free-space masks ($p_{c}^{\mathrm{occ}}<\tau_{\mathrm{free}}$) to constrain feasible trajectories.

### 3.3. Guidance Integration with Local Path Planning

To exploit MSCM-based cooperative guidance while preserving real-time autonomy, the vehicle executes a local path planning (LPP) module that generates dynamically feasible and guidance-compliant trajectories within its local map. The LPP operates on a Frenet coordinate representation, decoupling longitudinal and lateral motion along a reference path and enabling efficient optimization with external guidance cues.

The LPP comprises four stages:

1. Frenet Trajectory Generation. From the vehicle’s current Frenet state $\left(s_{0},d_{0},{\dot{s}}_{0},{\dot{d}}_{0}\right)$ and a region of interest (ROI) extracted from the HD map, we sample longitudinal *s* and lateral *d* offsets within bounds to form candidate trajectories $\Gamma =\{\gamma_{k}\}$. Each $\gamma_{k}$ is parameterized by quintic polynomials ensuring continuity in position, velocity, and acceleration.
2. Dynamic Feasibility Check. Candidates are filtered by kinematic/dynamic constraints (curvature, acceleration, jerk) and by the DOGM-based occupancy field (Section 3.2). Unsafe or infeasible paths are discarded.
3. Cost Evaluation. Surviving trajectories are scored by
  (11) $$J_{k}=\omega^{\mathrm{jerk}}J_{k}^{\mathrm{jerk}}+\omega^{\mathrm{risk}}J_{k}^{\mathrm{risk}}+\omega^{\mathrm{guide}}J_{k}^{\mathrm{guide}}.$$
  where the first term $J_{k}^{\mathrm{jerk}}$ penalizes lateral offset and smoothness; $J^{\mathrm{risk}}$ penalizes overlap with high-occupancy DOGM cells; and $J^{\mathrm{guide}}$ enforces compliance with MSCM (defined below). All three terms are normalized to comparable ranges prior to weighting, and $\left\{\omega^{\mathrm{jerk}},\omega^{\mathrm{risk}},\omega^{\mathrm{guide}}\right\}$ are selected to balance comfort, safety, and cooperation. This subjects the candidates to vehicle dynamics, comfort bounds (accel/jerk), collision-avoidance constraints from the fused perception, and guidance compliance from the cooperative maneuvering.
  Cooperative guidance from the MSCM can be encoded as soft penalties that are summed into the trajectory score $J_{k}^{\mathrm{guide}}$ in (8), and the cost function $J_{k}^{\mathrm{guide}}$ becomes
  (12) $$J_{k}^{\mathrm{guide}}=J_{k}^{\mathrm{lane}}+J_{k}^{\mathrm{speed}}+J_{k}^{\mathrm{time}}.$$
  Let $\gamma_{k}$ denote a candidate trajectory, $\gamma_{\mathrm{MSCM}}$ the reference lane/slot path implied by the received guidance, $v_{k}$ the trajectory speed profile, $\left[v_{\min},v_{\max}\right]$ the admissible bounds, $v_{\mathrm{ref}}$ the recommended speed, and $\left[t_{\mathrm{enter}},t_{\mathrm{leave}}\right]$ the admissible time window. Non-negative gains $\lambda_{\mathrm{lane}},\lambda_{v},\lambda_{t}$ tune the relative importance of spatial, speed, and timing compliance. Lane/slot adherence cost $J_{k}^{\mathrm{lane}}$, speed tracking and bound enforcement $J_{k}^{\mathrm{speed}}$, and time window compliance $J_{k}^{\mathrm{time}}$ are given by
  (13) $$\begin{array}{l} J_{k}^{\mathrm{lane}}=\lambda_{\mathrm{lane}}\;{∥\gamma_{k}-\gamma_{\mathrm{MSCM}}∥}^{2}, \end{array}$$
  (14) $$\begin{array}{cc} J_{k}^{\mathrm{speed}} & =\lambda_{v}\left[{\left(v_{k}-v_{\mathrm{ref}}\right)}^{2}+\chi_{v<v_{\min}}{\left(v_{\min}-v_{k}\right)}^{2}+\chi_{v>v_{\max}}{\left(v_{k}-v_{\max}\right)}^{2}\right], \end{array}$$
  (15) $$\begin{array}{cc} J_{k}^{\mathrm{time}} & =\lambda_{t}\;\phi \left(t_{k};t_{\mathrm{enter}},t_{\mathrm{leave}}\right), \end{array}$$
  where χ is defined as a binary penalty operator that activates only when the candidate speed profile $v_{k}$ violates the admissible bounds. Specifically, $\chi_{v<v_{\min}}=1$ if $v_{k}<v_{\min}$ and $\chi_{v>v_{\max}}=1$ if $v_{k}>v_{\max}$; otherwise, these terms evaluate to zero. The function $\phi (t_{k};t_{\mathrm{enter}},t_{\mathrm{leave}})$ represents a time window cost that quantifies the deviation of the trajectory timing $t_{k}$ from the guidance window $\left[t_{\mathrm{enter}},t_{\mathrm{leave}}\right]$, and is given by
  (16) $$\phi \left(t_{k};t_{\mathrm{enter}},t_{\mathrm{leave}}\right)=\left\{\begin{array}{cc} {\left(t_{\mathrm{enter}}-t_{k}\right)}^{2}, & \mathrm{if}\;t_{k}<t_{\mathrm{enter}}\\ 0, & \mathrm{if}\;t_{\mathrm{enter}}\leq t_{k}\leq t_{\mathrm{leave}}\\ {\left(t_{k}-t_{\mathrm{leave}}\right)}^{2}, & \mathrm{if}\;t_{k}>t_{\mathrm{leave}} \end{array}\right..$$
4. Selection and Tracking. The minimum cost trajectory $\gamma^{★}$ is selected and tracked by
  (17) $$\gamma^{★}=\arg\min_{\gamma_{k}\in \Gamma }J_{k}.$$
  The process repeats every control cycle (100 ms). If no feasible path satisfies cooperative constraints, the planner relaxes guidance costs and reverts to a safe OBS-only trajectory, in line with SAE J3216 fallback expectations.

### 3.4. MSCS-Based Intersection Management: FCFS vs. HPO

The MSCS enables vehicles and infrastructure to exchange near-term maneuver information so that participants can infer future driving intent and avoid conflicts proactively. Each vehicle transmits an MSCM with a planned trajectory (or path primitive), a speed profile, and an entry/exit time window for the conflict zone. Receivers—vehicles and/or the edge RSU—collect and compare these trajectories. If a conflict is detected, a vehicle can (i) adjust its own path or (ii) negotiate a revised intent by issuing an updated MSCM. Acting as a coordinator, the edge RSU can transmit guidance-bearing MSCMs to lower-priority vehicles (e.g., delayed entry time, adjusted speed, alternative slot/lane), which vehicles then incorporate as soft constraints in the local planner (Section 3.3).

A conflict arises when two MSCM trajectories overlap both (i) temporally within conflict zone time windows and (ii) spatially when vehicle footprints (with safety buffers) intersect along lane-level corridors. In practice, we use lane-pair conflict tables with conservative time windows and TTC/PET buffers for efficient screening [27].

#### 3.4.1. Baseline Policy: First-Come, First-Served (FCFS)

The vehicle that first arrives at a pre-entry reservation zone receives the right-of-way; remaining vehicles queue in First-In, First-Out (FIFO) order. Once the head-of-queue vehicle clears the conflict zone, the next vehicle is released. A reservation buffer is allocated upstream of the intersection. Upon entry, a vehicle is enqueued. The RSU issues an MSCM to the head-of-queue vehicle that encodes right-of-way, a nominal speed profile, and a time window through the conflict zone. FCFS is simple and predictable but conservative: it serializes flow even when mutually non-conflicting approaches could traverse concurrently.

#### 3.4.2. Proposed Policy: Hybrid Pairing Optimization (HPO)

Heuristic methods may not guarantee global optimality, but they can exploit structural opportunities that FCFS ignores. HPO augments FCFS with conflict-aware pairing: while preserving the earliest vehicle’s priority, the coordinator opportunistically admits additional vehicles whose MSCM trajectories are conflict-free with respect to the already admitted set. The proposed HPO is performed through the following steps:

(i) Approve the FCFS head vehicle.
(ii) Scan the queue in order and pair in any vehicle whose trajectory, footprint, or time window does not conflict with all already admitted vehicles.
(iii) For each admitted vehicle, issue a guidance-bearing MSCM specifying lane/slot, $\left[v_{\min},v_{\mathrm{ref}},v_{\max}\right]$, and $\left[t_{\mathrm{enter}},t_{\mathrm{leave}}\right]$.
(iv) If no conflict-free follower exists, HPO falls back to FCFS for that cycle.

The conflict predicate includes spatial overlap and time window separation with TTC/PET buffers. For queue length *N*, pairing is $O(N^{2})$ per cycle with lane–corridor pruning; in typical intersections, the short horizon keeps runtime modest.

## 4. Results and Performance Analysis

This section reports simulation results for the proposed edge RSU-enabled cooperative driving stack, focusing on (i) safety gains from SSS/SDSM at highway merges and (ii) efficiency gains from MSCS/MSCM-based cooperative maneuvering at unsignalized intersections and roundabouts. Unless noted otherwise, the primary metrics are average speed, delay, and intersection/roundabout throughput (veh/h). The vehicle stack employs the DOGM-based perception fusion in Section 3.2 and the Frenet local planner with guidance integration in Section 3.3. The cooperative operation flow follows Figure 2.

### 4.1. Simulation Setup

We evaluate three traffic scenes representative of edge RSU deployments: a highway merge with partial occlusions, an unsignalized four-way intersection, and a single-lane roundabout. In all scenes, CAVs execute the pipeline in Section 3 and exchange messages with the edge RSU over PC5. For perception experiments, we vary the SSS adoption rate among vehicles while keeping other parameters fixed. For maneuvering experiments, we compare the baseline FCFS and the proposed HPO coordinator policies (Section 3.4) under identical demand. Each scenario is simulated over multiple randomized seeds; we report mean trends. The simulation environment is implemented using MATLAB Automated Driving Toolbox and Driving Scenario Designer, with V2X message logic added to emulate edge–vehicle communication and infrastructure-guided CDA operations.

A potential concern is that our experiments were conducted within a MATLAB-based environment. We emphasize that the goal of this paper is not to claim absolute performance numbers tied to a specific simulator, but to quantify the algorithmic and architectural benefits of edge RSU-enabled CDA services when (i) message semantics follow SAE J3224 (SDSM) and SAE J3186 (MSCM) and (ii) the vehicle explicitly models internal–external fusion of cooperative perception/guidance with onboard sensing and planning. To support controlled, end-to-end comparisons (OBS-only vs. SSS; FCFS vs. HPO) under consistent timing and implementation assumptions, we implemented the full stack in a unified co-simulation environment composed of MATLAB Driving Scenario Designer, MATLAB Automated Driving Toolbox, and V2X message emulation. This single-platform integration reduces confounding factors introduced by multi-engine co-simulation, such as synchronization errors, coordinate frame mismatches, and heterogeneous time-step management, which can be nontrivial when coupling traffic simulators (e.g., SUMO) with 3D sensor/perception engines (e.g., CARLA).

Nevertheless, cross-platform replication remains important for assessing portability and robustness. We therefore interpret our findings as evidence of relative performance trends attributable to service design choices and fusion modeling: (i) infrastructure sensor sharing improves safety under occlusions by extending the effective sensing horizon; and (ii) maneuver guidance with lightweight conflict-aware pairing improves flow efficiency compared with strict FCFS under identical demand. Because the proposed fusion logic, cost shaping, and coordinator policies are formulated at the message/interface level (SDSM/MSCM) rather than being tied to simulator-specific APIs, they are largely platform-agnostic. As future work, we plan to reproduce representative scenarios using open-source stacks (e.g., SUMO+CARLA) and compare outcome trends under different sensor/rendering and traffic-flow implementations, including sensitivity to communication latency, packet loss, and message freshness.

### 4.2. Cooperative Perception at Highway Merges (SSS)

**Figure 3:**
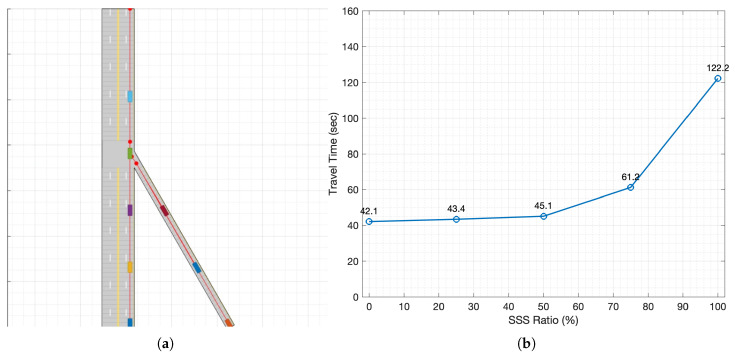
Highway merge scenario and impact of SSS adoption on efficiency. (**a**) Simulation layout of a single-lane on-ramp merging into the mainline; colored vehicles illustrate typical merge interactions near the conflict zone. (**b**) Mean travel time versus SSS penetration (0–100%).

A simulation was conducted in a highway merge environment to compare the driving safety of autonomous vehicles that use only OBS with those that use both OBS and SSS. Table 1 summarizes the parameters used in the scenario, and Figure 3a shows the highway merge environment in which the simulation was performed. The simulation uses a wrap-around method in which vehicles depart from the starting points of the main road and merging road. They pass through the merge point and reach the endpoint of the main road. Then, they return to the starting point to drive again. If a collision occurs at the merge point, the simulation ends. If no collision occurs and the vehicle completes five wrap-around cycles, the simulation ends and the duration is recorded as the travel time.

At low SSS adoption, vehicles relying primarily on OBS frequently reach unsafe states or collide in the merge zone, which in several runs prevents meaningful travel time statistics. When SSS is enabled, the edge RSU’s infrastructure perception extends the effective sensing horizon across occlusions; collision-free runs become prevalent, demonstrating a clear safety benefit.

A second observation concerns efficiency at high SSS penetration. To quantify the trend in Figure 3b, as the SSS penetration increases from 0% to 50%, the average travel time rises modestly from 42.1 s to 45.1 s. Between 50% and 75%, however, it jumps from 45.1 s to 61.2 s, and from 75% to 100% it nearly doubles again from 61.2 s to 122.2 s. This trend is not caused by an increase in the number of objects contained in SDSM. In our highway merge experiment, the total number of vehicles in the simulated world is fixed to $N_{\mathrm{veh}}=8$ throughout a run: vehicles are spawned from the mainline/ramp origins with an initial headway of 25 m and a cruising speed of 54 km/h, and they are re-inserted at the corresponding origin when reaching the end of the mainline (wrap-around). Therefore, the maximum number of dynamic vehicle objects that the edge RSU can perceive and include in each SDSM is upper-bounded by $N_{\mathrm{veh}}=8$, irrespective of the SSS penetration rate. We define the travel time as the duration required for all eight vehicles to complete five wrap-around cycles; this closed-system design prevents unbounded delay growth that would occur under open-boundary injection when congestion forms.

Instead, the travel time increase stems from how many vehicles utilize the same SDSM content in their onboard fusion and planning. Let $\rho_{\mathrm{SSS}}\in \left[0,1\right]$ denote the penetration rate and $V_{\mathrm{SSS}}$ the set of vehicles that fuse SDSM into the DOGM/LPP pipeline (Section 3.2 and Section 3.3); then $|V_{\mathrm{SSS}}|$ increases with $\rho_{\mathrm{SSS}}$ while the SDSM object cardinality remains bounded. As $\rho_{\mathrm{SSS}}$ grows, a larger fraction of vehicles internalizes infrastructure-detected occluded states early and expands high-risk regions in their local risk field. Because the merge scenario evaluates SSS alone (without MSCS-based intent negotiation or time window guidance), vehicles interpret potential conflicts conservatively to preserve safety margins, typically resulting in earlier deceleration, larger gap-acceptance thresholds, and cautious merging decisions. When only a small subset of vehicles uses SSS, this conservative behavior is localized; however, at high penetration, it becomes system-wide and amplifies the bottleneck at the merge point, which increases the average cycle time and, consequently, the measured travel time.

These results suggest a service-mix implication: SSS provides clear safety benefits under occlusion, but high-penetration deployments may require complementary maneuver guidance (e.g., MSCS/MSCM) or adaptive fusion/planning policies to mitigate overly conservative behavior while maintaining safety.

### 4.3. Cooperative Maneuvering at Unsignalized Intersections and Roundabouts (MSCS)

**Figure 4:**
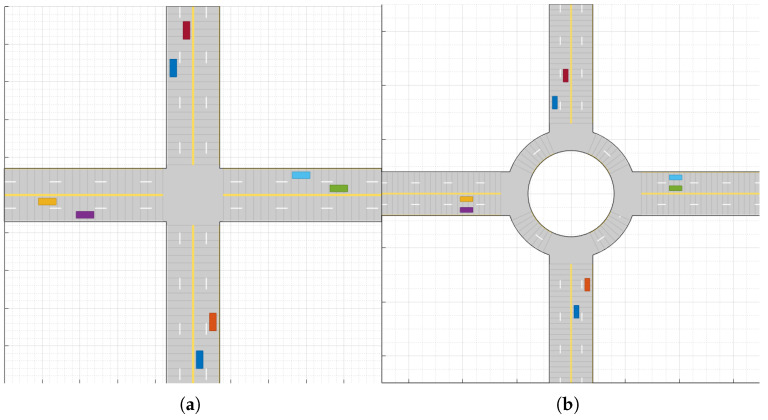
Simulation layouts for cooperative maneuvering experiments. (**a**) Unsignalized four-leg intersection with two-lane approaches. (**b**) Four-approach single-lane roundabout.

To analyze driving efficiency improvements achieved through MSCS, simulations were conducted in both unsignalized intersections and roundabouts. Table 1 shows the parameters used in the scenarios, while Figure 4 illustrates the structures of the unsignalized intersection and roundabout where simulations were performed. The simulation runs for 100 seconds using a wrap-around method, where CAVs start driving from the beginning point of the forward-direction road, continue driving until reaching the end point, and then return to the starting point to drive again.

We compare FCFS with the proposed HPO at an unsignalized intersection. FCFS grants right-of-way strictly by arrival order to a pre-entry buffer, yielding predictable but serialized service. HPO preserves FCFS priority for the earliest vehicle and pairs in additional followers whose MSCM trajectories do not overlap in time/space with already-admitted paths. Across seeds, HPO consistently increases average speed and increases throughput relative to FCFS.

**Figure 5:**
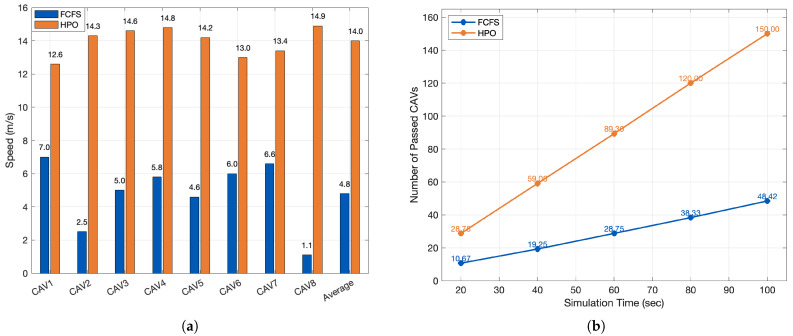
Performance comparison between the baseline FCFS and the proposed HPO at an unsignalized intersection. (**a**) Per-vehicle and average speed (m/s). (**b**) Cumulative number of passed CAVs over time.

Quantitatively, the bar graph in Figure 5a shows that the average speed with HPO is 14.0 m/s versus 4.8 m/s under FCFS, i.e., a +192% improvement. The per-vehicle speeds also exhibit a tighter band with HPO (12.6–14.9 m/s) than with FCFS (1.1–7.0 m/s), indicating a more stable progression between vehicles. Consistent trends appear in Figure 5b: by 100 s, the cumulative number of passed CAVs reaches 150 with HPO compared to 48.42 with FCFS. The average throughput slope over 20 s intervals is ≈1.49 veh/s for HPO versus ≈0.47 veh/s for FCFS, confirming that HPO sustains substantially higher discharge rates while preserving the safety constraints enforced by the pairing test.

The roundabout yields the same qualitative behavior but with larger gains than the unsignalized intersection. Because vehicles traverse a longer conflict corridor, HPO finds more opportunities to admit additional, non-interfering flows, delivering substantially higher throughput and higher mean speeds than FCFS.

**Figure 6:**
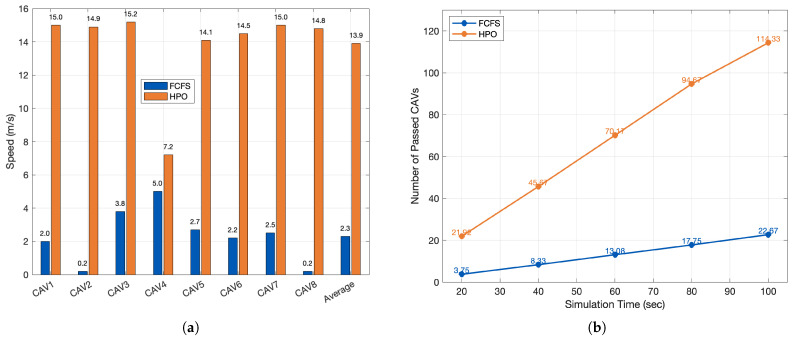
Performance comparison between the baseline FCFS and the proposed HPO at a roundabout. (**a**) Per-vehicle and average speed (m/s). (**b**) Cumulative number of passed CAVs over time.

Quantitatively, Figure 6a shows that the mean speed under HPO is 13.9 m/s versus 2.3 m/s with FCFS—an absolute gain of +11.6 m/s. Per-vehicle dispersion is also much tighter with HPO (mostly 14–15 m/s, one outlier at 7.2 m/s) than with FCFS (0.2–5.0 m/s), indicating more uniform progression across CAVs. Consistently, Figure 6b reports that by 100 s, the cumulative number of passed CAVs reaches 114.33 with HPO but only 22.67 with FCFS. Over each 20 s interval, the average discharge increment is ≈1.18 veh/s for HPO versus ≈0.24 veh/s for FCFS—again about a five-fold throughput advantage while maintaining the safety checks imposed by the pairing test.

The results suggest a service-mix insight: (i) SSS is highly effective at improving safety in occlusion-prone merges but, by itself, can be conservative for efficiency at high penetration; and (ii) MSCS (with HPO) provides strong efficiency gains in conflict zones while preserving safety through advisory windows and continuous revalidation.

While the simulation results show consistent gains from SSS and MSCS policies, they are derived under controlled environments with idealized sensor models and perfect communication. Real-world deployment would face additional uncertainties such as non-V2X actors, packet loss, and localization errors. Future work includes validating our pipeline in a hardware-in-the-loop setting to assess robustness and generalizability.

The three scenarios represent distinct conflict geometries and thus stress different aspects of edge-enabled CDA. In highway merging, the dominant failure mode is perception-limited decision-making under partial occlusions; accordingly, SSS/SDSM primarily improves safety by expanding the effective sensing horizon and reducing NLOS-induced uncertainty. In contrast, at unsignalized intersections and roundabouts, the dominant bottleneck is spatiotemporal conflict resolution among multiple agents; here, MSCS/MSCM guidance primarily improves efficiency by shaping feasible time windows and reducing deadlocks while maintaining arrival order fairness. To isolate these service-specific effects, we held environmental factors (e.g., sensor noise model and road friction) constant and varied only the SSS penetration rate in the merge scenario and the coordinator policy (FCFS vs. HPO) under identical demand in intersection/roundabout scenarios. We acknowledge that broader sensitivity analysis over traffic density, heterogeneous penetration rates, and adverse sensing/communication conditions is important for generalization; we therefore add these dimensions to our future-work roadmap in Section 5.

## 5. Discussion and Deployment Considerations

### 5.1. From Simulation Results to Real-World Deployment

Our results quantify the relative benefits of edge RSU-enabled SSS (safety) and MSCS guidance (safety and efficiency) under controlled assumptions. When translating these findings to real deployments, three practical constraints are particularly important.

(i) Communication range and service area. In practice, the effective service area is constrained by PC5 coverage, antenna placement, and roadside clutter. Accordingly, edge RSUs should be deployed at well-defined conflict zones (e.g., merges, unsignalized intersections, roundabouts), and the cooperative services should be activated within a bounded geofence where (a) V2X connectivity is reliable and (b) infrastructure sensors provide sufficient line-of-sight coverage.
(ii) End-to-end latency and staleness. Safety and coordination gains depend on the timeliness of cooperative information, which is affected by sensing, edge computation, and wireless transmission. Our vehicle-side fusion explicitly accounts for message staleness via AoI-aware down-weighting, and thus provides a principled mechanism to degrade gracefully when updates are delayed. In deployment, latency budgets should be engineered jointly across sensing, MEC processing, and sidelink scheduling, and AoI statistics can be monitored online to adapt service rates and fusion weights.
(iii) Integration with existing traffic infrastructure. Edge RSUs can be integrated with existing roadside assets (e.g., traffic cabinets, power/backhaul, and MAP/SPaT infrastructure) to reduce installation cost and enable tighter coupling with traffic management. In particular, the same edge platform can host (a) local perception services for CDA, (b) interfaces to conventional ITS components, and (c) logging/monitoring functions for safety auditing.

### 5.2. Scenario Sensitivity: Density, Environment, and Penetration Rates

The scenarios in this paper are intended as representative building blocks for edge RSU deployments, rather than an exhaustive coverage of all traffic and environmental conditions. We partially address penetration rate sensitivity by sweeping the SSS adoption rate in the merge scenario, which reveals non-monotonic behavior in travel time at high penetration due to system-level interactions between cooperative perception and local planning. For intersections and roundabouts, we compare FCFS and HPO under identical demand to isolate the impact of guidance generation and injection. Future work will extend the study along three axes: (i) broader traffic densities and mixed traffic compositions (e.g., human driving vehicles and CAVs), (ii) adverse sensing conditions (e.g., rain/fog and degraded detection quality), and (iii) heterogeneous and time-varying penetration of edge-enabled services, including partial availability of SSS vs. MSCS.

### 5.3. Security and Trust Considerations (Qualitative)

Because edge RSUs can influence vehicle perception and maneuver selection, the communication and computation chain must be protected against spoofing, replay, and data manipulation. At a minimum, edge-to-vehicle cooperative messages should be authenticated and integrity-protected, and the edge platform should support secure key storage and trusted execution for message generation. In addition to cryptographic protection, plausibility checks at the vehicle (e.g., consistency between onboard observations and received SDSM/MSCM) and misbehavior detection at the infrastructure can limit the impact of compromised nodes. While a full security evaluation is beyond the scope of this paper, we add this discussion to clarify deployment implications and to motivate future work on secure edge-assisted CDA.

### 5.4. Future Work: 5G/Next-Generation V2X and ITS Integration

Emerging V2X capabilities can further strengthen edge RSU-assisted CDA. First, 5G NR-V2X evolution can improve reliability and reduce latency variability, enabling tighter AoI control and more frequent cooperative updates. Second, edge orchestration mechanisms (e.g., dynamic compute placement and service scaling) can adapt cooperative services to congestion and workload variations. Finally, integrating edge RSU-enabled CDA with broader ITS functions (e.g., traffic signal control, digital-twin monitoring, and corridor-level traffic management) can enable system-wide optimization while preserving local safety constraints at conflict zones.

## 6. Conclusions

We presented an edge RSU architecture that generates SSS-compliant SDSM (infrastructure cooperative perception) and MSCS-style guidance (cooperative maneuvering), together with a vehicle-side fusion pipeline that integrates these artifacts with onboard sensing and Frenet planning. Simulations across highway merges, unsignalized intersections, and roundabouts show the following:

- Cooperative perception (SSS) markedly improves safety in occlusion-heavy merges by reducing collisions and unsafe states relative to OBS-only autonomy.
- Cooperative maneuvering (MSCS)—particularly the proposed HPO pairing policy—increases average speed and throughput versus FCFS at intersections and roundabouts while preserving safety via time window and corridor checks.
- The combination of SSS and MSCS strikes a favorable safety–efficiency balance, provided that message freshness and integrity are enforced and vehicle-side costs are scaled accordingly.

Future work will extend the framework in three directions. First, we will explore joint SSS–MSCS policies at merges (e.g., intent-aware gap creation) to mitigate the efficiency loss observed with perception-only sharing. Second, we will perform a latency/AoI sensitivity sweep using measured PC5/MEC delays and controlled packet losses to quantify robustness margins. Third, we are planning a hardware-in-the-loop pilot with real RSU sensors and CAVs to validate DOGM fusion and HPO pairing in the field, including mixed traffic with non-V2X road users.

Overall, the results support the deployability of edge RSU-enabled CDA: SSS offers immediate safety benefits in challenging visibility conditions, while MSCS—implemented with lightweight heuristics such as the proposed HPO—unlocks meaningful efficiency gains in routine intersection and roundabout operations. The proposed architecture supports a modular upgrade path for existing infrastructure and automated vehicle stacks, promoting gradual CDA deployment.

## Figures and Tables

**Table 1:** Simulation parameters used in the two scenarios.

Parameter	Highway Merge (SSS)	Unsignalized Intersection / Roundabout (MSCS)
Simulation duration	100 s	*∞* (open-ended)
Time resolution	100 ms	100 ms
Vehicle footprint	4.7×1.8 m (length × width)	4.7×1.8 m (length × width)
Safety buffer	1.2× vehicle footprint (120%)	1.2× vehicle footprint (120%)
Cruising speed	54 km/h	90 km/h
Acceleration limit	15 m/$s^{2}$	15 m/$s^{2}$
Initial inter-vehicle gap	25 m	25 m
Number of sensors	2 (one front, one rear)	2 (one front, one rear)
Sensor range	50 m	50 m
Sensor field-of-view	$\pm 45^{\circ }$	$\pm 45^{\circ }$
Lane width	3.5 m	3.5 m

## Data Availability

The original contributions presented in this study are included in the article. Further inquiries can be directed to the corresponding author.
